# 2-{2-[5-(4-Cyano-5-di­cyano­methyl­idene-2,2-dimethyl-2,5-di­hydro­furan-3-yl)penta-2,4-dienyl­idene]-3,3-dimethyl-2,3-di­hydro-1*H*-indol-1-yl}ethyl 3,5-bis­(benz­yloxy)benzoate

**DOI:** 10.1107/S1600536813032868

**Published:** 2013-12-11

**Authors:** Graeme J. Gainsford, M. Delower H. Bhuiyan, Andrew J. Kay

**Affiliations:** a Callaghan Innovation, PO Box 31-310, Lower Hutt, 5040, New Zealand

## Abstract

In the title mol­ecule, C_48_H_42_N_4_O_5_, a potential non-linear optical compound, the furan ring [r.m.s. deviation = 0.010 (1) Å] and the indolyl­idene ring system [r.m.s. deviation = 0.013 (2) Å] are inclined to one another by 18.52 (6)°. This is similar to the arrangement [16.51 (18)°] found for the *N*-hy­droxy­ethyl adduct of the title compound [Bhuiyan *et al.* (2011 ▶). *Mol. Cryst. Liq. Cryst.* **548**, 1–12]. Replacing the hy­droxy­ethyl group with 3,5-di­benzyl­oxybenzoate has not resulted in a non-centrosymmetric lattice arrangement or significant changes to the basic mol­ecular structure. In the crystal, mol­ecules are linked *via* pairs of C—H⋯N hydrogen bonds, forming inversion dimers with an *R* ^2^_2_(20) ring motif. The dimers are linked *via* C—H⋯O hydrogen bonds, forming *C*(17) chains along [010]. The chains are linked by further C—H⋯N hydrogen bonds, forming layers parallel to (001) and enclosing *R*_2_^2^(44) ring motifs. There are also C—H⋯π inter­actions present, stabilizing the inter­layer orientation of the pendant bis­(benz­yloxy)benzo­yloxy group.

## Related literature   

For general background to organic non-linear optical (NLO) materials and details of similar structures, see: Kim *et al.* (2007 ▶); Gainsford *et al.* (2007 ▶, 2008 ▶); Smith *et al.* (2006 ▶); Bhuiyan *et al.* (2011 ▶); Li *et al.* (2005 ▶); Ojala *et al.* (2012 ▶). For the synthesis of the title compound, see: Clarke *et al.* (2009 ▶). For details of the *N*-hy­droxy­ethyl adduct of the title compound, see: Bhuiyan *et al.* (2011 ▶). For hydrogen-bond motifs, see: Bernstein *et al.* (1995 ▶). For details of the Cambridge Structural Database (CSD), see: Allen (2002 ▶).
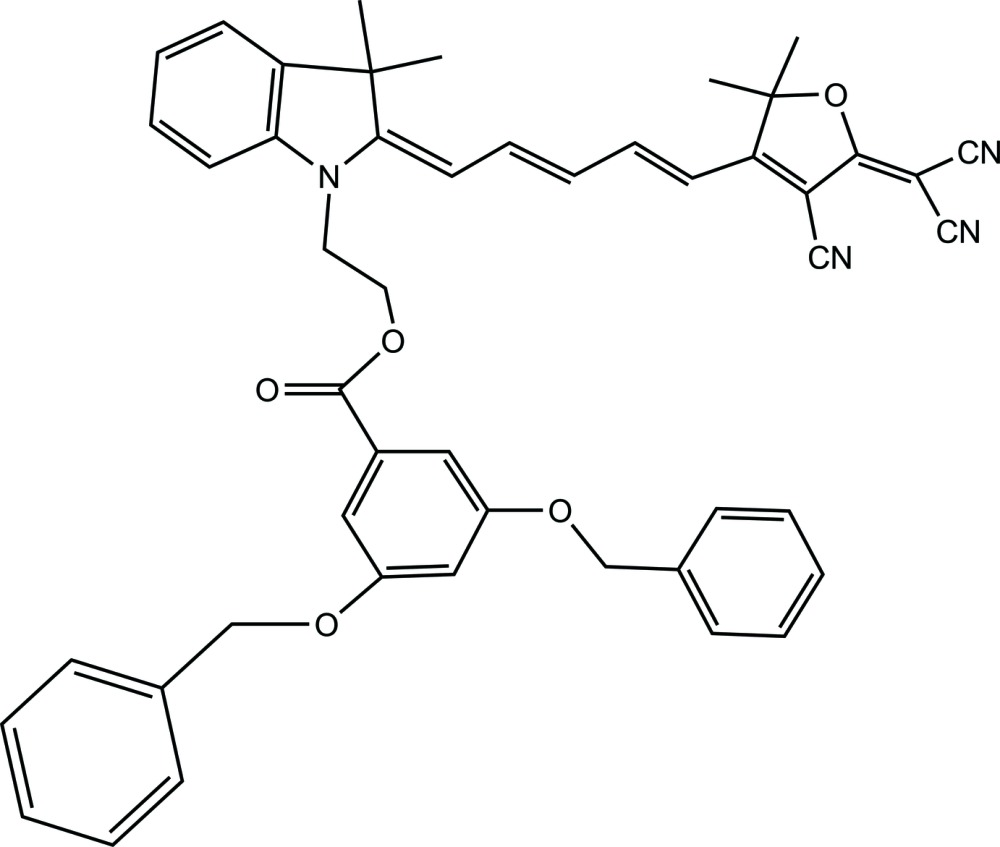


## Experimental   

### 

#### Crystal data  

C_48_H_42_N_4_O_5_
*M* *_r_* = 754.86
Monoclinic, 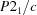

*a* = 16.1925 (5) Å
*b* = 15.6802 (5) Å
*c* = 17.6529 (6) Å
β = 115.038 (2)°
*V* = 4060.9 (2) Å^3^
*Z* = 4
Mo *K*α radiation
μ = 0.08 mm^−1^
*T* = 120 K
0.31 × 0.26 × 0.25 mm

#### Data collection  

Bruker APEXII CCD diffractometer
Absorption correction: multi-scan (*SORTAV*; Blessing, 1995 ▶) *T*_min_ = 0.682, *T*_max_ = 0.746
106328 measured reflections
14582 independent reflections
9621 reflections with *I* > 2σ(*I*)
*R*_int_ = 0.049

#### Refinement  

*R*[*F* ^2^ > 2σ(*F* ^2^)] = 0.054
*wR*(*F* ^2^) = 0.153
*S* = 1.02
14582 reflections
518 parameters
H-atom parameters constrained
Δρ_max_ = 0.48 e Å^−3^
Δρ_min_ = −0.25 e Å^−3^

### 

Data collection: *APEX2* (Bruker, 2005 ▶); cell refinement: *SAINT* (Bruker, 2005 ▶); data reduction: *SAINT*; program(s) used to solve structure: *SHELXS97* (Sheldrick, 2008 ▶); program(s) used to refine structure: *SHELXL97* (Sheldrick, 2008 ▶); molecular graphics: *ORTEP-3 for Windows* (Farrugia, 2012 ▶) and *Mercury* (Macrae *et al.*, 2008 ▶); software used to prepare material for publication: *SHELXL97* and *PLATON* (Spek, 2009 ▶).

## Supplementary Material

Crystal structure: contains datablock(s) global, I. DOI: 10.1107/S1600536813032868/su2670sup1.cif

Structure factors: contains datablock(s) I. DOI: 10.1107/S1600536813032868/su2670Isup2.hkl

Supporting information file. DOI: 10.1107/S1600536813032868/su2670Isup3.cml

Additional supporting information:  crystallographic information; 3D view; checkCIF report

## Figures and Tables

**Table 1:** Hydrogen-bond geometry (Å, °) *Cg*1 is the centroid of the C29–C34 ring.

*D*—H⋯*A*	*D*—H	H⋯*A*	*D*⋯*A*	*D*—H⋯*A*
C15—H15⋯N3^i^	0.95	2.48	3.404 (2)	165
C30—H30⋯O1^ii^	0.95	2.50	3.4136 (17)	162
C32—H32⋯N2^iii^	0.95	2.54	3.475 (2)	167
C9—H9*A*⋯*Cg*1^iii^	0.98	2.89	3.8454 (16)	166

Symmetry codes: (i) 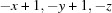; (ii) 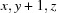; (iii) 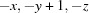.

**Fig. 1.**
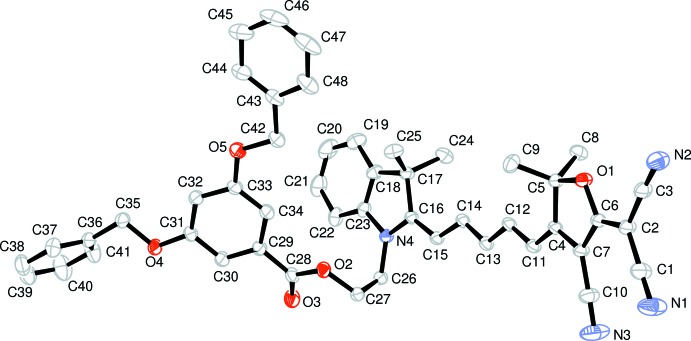
Molecular structure of the title molecule, with atom labelling. Displacement ellipsoids are drawn at the 50% probability level. H atoms have been omitted for clarity.

**Fig. 2.**
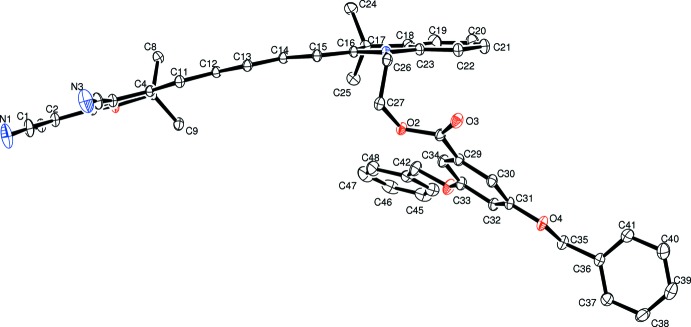
Edge-on view of the title molecule illustrating the molecular twisting from planarity and relative conformations.

**Fig. 3.**
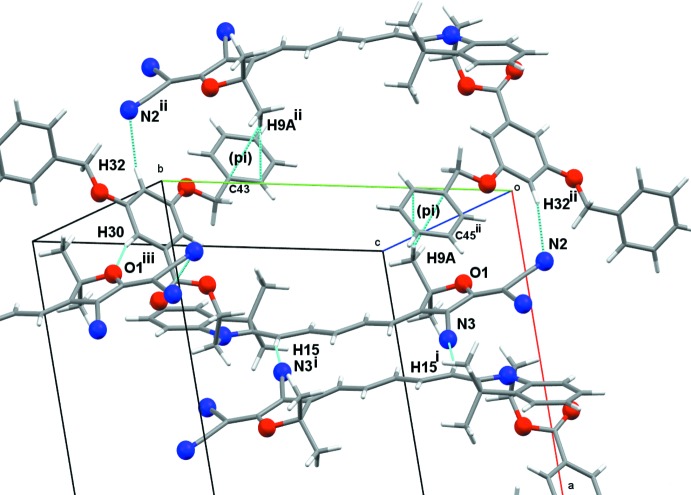
A partial view of the crystal packing of the title compound. The hydrogen bonds are shown as dashed lines (see Table 1 for details).

## supplementary crystallographic information

### 1. Comment

Organic non-linear optical (NLO) chromophores are highly polar and tend to readily form aggregates in both solution and/or the solid state (Smith *et al.*, 2006). This is a potential downfall when considering the usage of NLO materials in a host polymer. The presence of aggregation will lower the overall poling efficiency and increase the tendency for relaxation of the aligned dipoles which decreases the observed macroscopic response. The introduction of bulky, arene-rich substituents has been shown to be very effective in reducing aggregation and increasing the observed NLO response (Kim *et al.*, 2007). We report herein on the synthesis of an indoline chromophore which contains a 3,5-dibenzyloxybenzoate substituent and which was designed to reduce the tendency for molecular aggregation to occur.

A number of related compounds, namely 2-(3-Cyano-4-(3-(1-decyl-1,4-dihydroquinolin-4-ylidene)prop-1-enyl)-5,5-dimethyl- 2,5-dihydrofuran-2-ylidene)malononitrile (NOJKUT; Gainsford *et al.*, 2008), (4-Butyl-5-(2-(1-butyl-3,3-dimethyl-1,3-dihydro-2H-indol-2-ylidene)ethylidene)-1,3-thiazol-2(5H)-ylidene)malononitrile (NAPZAH; Ojala *et al.*, 2012) and 2-(4-(4-(N-Formylanilino)-trans-1,3-butadienyl)-3-cyano-5,5-dimethyl- 2,5-dihydrofuranylidene)propanedinitrile (GIMQAV; Gainsford *et al.*, 2007) were located in the Cambridge Structural Database (CSD; V5.34, last update May 2013; Allen, 2002).

The molecular structure of the title compound is shown in Fig. 1. The 5-membered ring plane of atoms (O1/C4—C7) of the acceptor group (hereafter CTF; 3-cyano-5,5-Dimethyl-2,5-dihydrofuran-2-ylidene]propanedinitrile) can be regarded as planar [r.m.s. deviations 0.010 (1) Å]. The dicyano group (N1/C1-C3/N2) is planar [r.m.s.d. 0.013 (2) Å] but twisted by 5.75 (10)° with respect to the CTF group; this is similar to the twist in the related compound (NOJKUT - see above) of 5.69 (17)°. We note that in the related compound (NAPZAH - see above) the subtended dicyano group is coplanar with the 1,3-thiazolylidene ring.

The fused indolylidene system (N4/C16-C23) is also essentially planar [r.m.s.d. 0.013 (2) Å] and makes a dihedral angle with the CTF ring of 18.52 (6)°, similar to the 16.51 (18)° angle found in the *N*-hydroxyethyl adduct of the title compound, 2-(3-cyano-4-{5-[1-(2-hydroxy-ethyl)-3,3-dimethyl-1,3-dihydro- indol-2-ylidene]-penta-1,3-dienyl}-5,5-dimethyl-5*H*-furan-2-ylidene)-malononitrile (henceforth FAFP; Bhuiyan *et al.*, 2011). This angle reflects a twist in the C11–C14 polyene chain beginning at C11 and the plane through C11–C14 subtends 7.23 (13)° with the CTF plane; a view illustrating the relative conformations of the various chemical entities is given in Fig. 2. Again this is in contrast to the smaller NAPZAH structure where the polyene chain atoms and indolylidene ring are coplanar, and twist from the 5-membered 1,3-thiazolylidene ring plane by 5.48 (6)°. Rings A (C29–C34) and B (C43–C48) subtend an angle of 18.72 (7)°, whilst the phenyl ring C (C36–C41) makes an angle of 54.69 (8)° to ring A, and 65.37 (9)° to ring B. Ring A makes an angle of 44.54 (6)° to the indolylidene ring.

There is considerable delocalization of charge along the polyene/CTF chain with a bond length alternation (BLA) value of 0.016 Å compared with the free CTF value of 0.108 Å (Li *et al.*, 2005) 0.060 Å in (GIMQAV - see above) and 0.024 Å in FAFP.

The crystal packing involves attractive non-classical hydrogen bond interactions of the (alkene)*C*—*H*···*N*(cyano), (phenyl)C—H···O and phenyl(*C*—*H*)···*N*(cyano) types (Table 1, Fig. 3). The alkene H15···N3 interaction (entry 1, Table 1) connects molecules around centers of symmetry (*e.g.* at 1/2, 1/2, 0) into dimer layers, approximately parallel to (1,-1,1) or (3,1,1) crystallographic planes, which can be described by the H bonding motif *R*^2^_2_(20) (Bernstein *et al.*, 1995). The other two main contacts (entries 2 and 3, Table 1) connect other molecules into these layers. The H30···O1 interaction forms a C(17) motif as it links the identical molecule related by a *b* axis translation. The H32···N2 interactions form a *R*^2^_2_(44) motif utilizing an inversion center at (0, 1/2, 0). In addition, there are C—H···π interactions between methyl H9A and the phenyl ring (atoms C43—C48) which stabilizes the interlayer orientation of the "dangling" bis-benzyloxoy-benzoic acid moiety. Providing weak links between the layers are alkene *C*—*H*···*N*(cyano) interactions involving atoms C12 and C14, an interaction also observed previously in FAFP (Bhuiyan *et al.*, 2011).

### 2. Experimental

The title compound was synthesized by the procedure described by Clarke *et al.* (2009). Single crystals were grown by slow ether diffusion into an ethyl acetate solution of the title compound. Spectroscopic and other data for the title compound are included in the archived CIF.

### 3. Refinement

Eight reflections affected by the backstop were omitted from the refinement. All the H atoms were placed in geometrically idealized positions and constrained to ride on their parent atoms: C—H = 0.98, 0.99 and 0.95 Å CH_3_, CH_2_ and CH(aromatic) H atoms, respectively, with *U*_iso_(H) = 1.5*U*_eq_(C-methyl) and = 1.2U_eq_(C) for other H atoms. The methyl H atoms were allowed to rotate freely about the adjacent C—C bond.

### Crystal data

**Table:**

C_48_H_42_N_4_O_5_	*F*(000) = 1592
*M**_r_* = 754.86	*D*_x_ = 1.235 Mg m^−^^3^
Monoclinic, *P*2_1_/*c*	Mo *K*α radiation, λ = 0.71073 Å
Hall symbol: -P 2ybc	Cell parameters from 9867 reflections
*a* = 16.1925 (5) Å	θ = 2.6–31.1°
*b* = 15.6802 (5) Å	µ = 0.08 mm^−^^1^
*c* = 17.6529 (6) Å	*T* = 120 K
β = 115.038 (2)°	Block, blue
*V* = 4060.9 (2) Å^3^	0.31 × 0.26 × 0.25 mm
*Z* = 4	

### Data collection

**Table:**

Bruker APEXII CCD diffractometer	14582 independent reflections
Radiation source: fine-focus sealed tube	9621 reflections with *I* > 2σ(*I*)
Graphite monochromator	*R*_int_ = 0.049
Detector resolution: 8.333 pixels mm^-1^	θ_max_ = 32.7°, θ_min_ = 2.6°
φ and ω scans	*h* = −24→24
Absorption correction: multi-scan (*SORTAV*; Blessing, 1995)	*k* = −23→23
*T*_min_ = 0.682, *T*_max_ = 0.746	*l* = −26→25
106328 measured reflections	

### Refinement

**Table:**

Refinement on *F*^2^	Primary atom site location: structure-invariant direct methods
Least-squares matrix: full	Secondary atom site location: difference Fourier map
*R*[*F*^2^ > 2σ(*F*^2^)] = 0.054	Hydrogen site location: inferred from neighbouring sites
*wR*(*F*^2^) = 0.153	H-atom parameters constrained
*S* = 1.02	*w* = 1/[σ^2^(*F*_o_^2^) + (0.0778*P*)^2^ + 0.7163*P*] where *P* = (*F*_o_^2^ + 2*F*_c_^2^)/3
14582 reflections	(Δ/σ)_max_ = 0.001
518 parameters	Δρ_max_ = 0.48 e Å^−^^3^
0 restraints	Δρ_min_ = −0.25 e Å^−^^3^

### Special details

**Table:**

Experimental. Spectroscopic and other data for the title compound: ^1^H NMR (d_6_-DMSO) δ 1.60 (6H, s, 2xCH3), 1.64 (6H, s, 2xCH3), 4.51 (2H, t, J 5.1 Hz, CH2), 4.65 (2H, t, J 5.1, CH2), 5.02 (4H, s, 2xCH2), 6.01 (1H, d, J 14.2 Hz, CH), 6.32–6.43 (2H, m, 2xCH), 6.86 (1H, t, J 2.3 Hz, ArH), 7.05 (2H, d, J 2.5 Hz, ArH), 7.15 (1 H, t, J 8.0 Hz, ArH), 7.29–7.41 (11H, m, ArH), 7.45 (1H, d, J 8.0 Hz, ArH), 7.54 (1H, d, J 7.6 Hz, ArH), 7.82 (1H, t, J 13.5 Hz, CH), 8.06 (1H, t, J 13.5 Hz, CH). ^13^C NMR (d_6_-DMSO) δ 26.8, 27.5, 42.9, 45.9, 48.9, 61.9, 69.9, 95.9, 103.8, 107.5, 108.4, 109.2, 111.2, 113.5, 114.5, 115.3, 122.6, 124.6, 125.9, 127.6, 128.3, 128.8, 131.4, 136.8, 141.0, 142.6, 151.7, 152.8, 159.7, 165.5, 170.8, 172.1, 176.8. MS - Found: MNa^+^ 777.3050; Calc: 777.3053; Δ = 0.3 p.p.m.; M.p. = 549 K (dec.).
Geometry. All e.s.d.'s (except the e.s.d. in the dihedral angle between two l.s. planes) are estimated using the full covariance matrix. The cell e.s.d.'s are taken into account individually in the estimation of e.s.d.'s in distances, angles and torsion angles; correlations between e.s.d.'s in cell parameters are only used when they are defined by crystal symmetry. An approximate (isotropic) treatment of cell e.s.d.'s is used for estimating e.s.d.'s involving l.s. planes.
Refinement. Refinement of *F*^2^ against ALL reflections. The weighted *R*-factor *wR* and goodness of fit *S* are based on *F*^2^, conventional *R*-factors *R* are based on *F*, with *F* set to zero for negative *F*^2^. The threshold expression of *F*^2^ > σ(*F*^2^) is used only for calculating *R*-factors(gt) *etc*. and is not relevant to the choice of reflections for refinement. *R*-factors based on *F*^2^ are statistically about twice as large as those based on *F*, and *R*- factors based on ALL data will be even larger.

### Fractional atomic coordinates and isotropic or equivalent isotropic displacement parameters (Å^2^)

**Table:**

	*x*	*y*	*z*	*U*_iso_*/*U*_eq_	
O1	0.27488 (6)	0.17454 (5)	0.02308 (5)	0.02415 (17)	
O2	0.28317 (6)	0.87522 (5)	0.11107 (6)	0.02664 (18)	
O3	0.33004 (7)	1.01185 (6)	0.13326 (7)	0.0354 (2)	
O4	0.02801 (6)	1.15735 (5)	0.06642 (6)	0.0315 (2)	
O5	−0.00979 (7)	0.87452 (6)	0.14487 (7)	0.0380 (2)	
N1	0.36962 (14)	0.09712 (9)	−0.18815 (11)	0.0610 (5)	
N2	0.21981 (9)	−0.03146 (8)	−0.07112 (9)	0.0389 (3)	
N3	0.46305 (13)	0.29291 (10)	−0.09615 (11)	0.0584 (4)	
N4	0.40465 (7)	0.79660 (6)	0.25765 (6)	0.02294 (19)	
C1	0.34424 (12)	0.10072 (8)	−0.13685 (10)	0.0375 (3)	
C2	0.30870 (9)	0.10250 (7)	−0.07603 (8)	0.0266 (2)	
C3	0.25862 (9)	0.02976 (8)	−0.07198 (8)	0.0281 (2)	
C4	0.35515 (8)	0.30273 (7)	0.04015 (7)	0.0212 (2)	
C5	0.29468 (8)	0.25456 (7)	0.07175 (7)	0.0213 (2)	
C6	0.31764 (8)	0.17354 (7)	−0.02671 (7)	0.0221 (2)	
C7	0.36677 (9)	0.25051 (7)	−0.01856 (8)	0.0234 (2)	
C8	0.34273 (9)	0.22813 (8)	0.16286 (8)	0.0272 (2)	
H8A	0.3048	0.1871	0.1757	0.041*	
H8B	0.3531	0.2785	0.1986	0.041*	
H8C	0.4014	0.2017	0.1731	0.041*	
C9	0.20331 (9)	0.29729 (8)	0.04958 (9)	0.0306 (3)	
H9A	0.1762	0.3130	−0.0097	0.046*	
H9B	0.2120	0.3486	0.0837	0.046*	
H9C	0.1626	0.2578	0.0604	0.046*	
C10	0.41958 (11)	0.27260 (8)	−0.06229 (9)	0.0337 (3)	
C11	0.39111 (9)	0.38450 (7)	0.06261 (7)	0.0236 (2)	
H11	0.4262	0.4064	0.0354	0.028*	
C12	0.38020 (8)	0.43656 (7)	0.12114 (7)	0.0225 (2)	
H12	0.3501	0.4133	0.1524	0.027*	
C13	0.41038 (8)	0.52084 (7)	0.13727 (7)	0.0233 (2)	
H13	0.4431	0.5446	0.1086	0.028*	
C14	0.39374 (8)	0.57040 (7)	0.19393 (7)	0.0229 (2)	
H14	0.3681	0.5438	0.2274	0.028*	
C15	0.41262 (8)	0.65764 (7)	0.20468 (7)	0.0227 (2)	
H15	0.4441	0.6821	0.1752	0.027*	
C16	0.38935 (8)	0.71132 (7)	0.25470 (7)	0.0217 (2)	
C17	0.34510 (9)	0.68940 (8)	0.31372 (8)	0.0262 (2)	
C18	0.33633 (10)	0.77700 (9)	0.34542 (8)	0.0307 (3)	
C19	0.29889 (12)	0.80185 (11)	0.39941 (10)	0.0450 (4)	
H19	0.2734	0.7609	0.4231	0.054*	
C20	0.29939 (14)	0.88811 (12)	0.41813 (11)	0.0526 (4)	
H20	0.2745	0.9062	0.4555	0.063*	
C21	0.33549 (12)	0.94761 (11)	0.38333 (10)	0.0453 (4)	
H21	0.3352	1.0061	0.3973	0.054*	
C22	0.37253 (10)	0.92416 (9)	0.32799 (9)	0.0337 (3)	
H22	0.3969	0.9652	0.3035	0.040*	
C23	0.37188 (9)	0.83781 (8)	0.31062 (8)	0.0265 (2)	
C24	0.40886 (10)	0.63264 (9)	0.38589 (8)	0.0343 (3)	
H24A	0.4681	0.6608	0.4145	0.052*	
H24B	0.4171	0.5777	0.3635	0.052*	
H24C	0.3820	0.6233	0.4256	0.052*	
C25	0.25050 (10)	0.64853 (9)	0.26807 (9)	0.0343 (3)	
H25A	0.2206	0.6455	0.3060	0.051*	
H25B	0.2571	0.5909	0.2499	0.051*	
H25C	0.2135	0.6831	0.2192	0.051*	
C26	0.44046 (8)	0.84259 (7)	0.20598 (8)	0.0241 (2)	
H26A	0.4949	0.8123	0.2076	0.029*	
H26B	0.4598	0.9003	0.2295	0.029*	
C27	0.37088 (9)	0.85040 (7)	0.11610 (8)	0.0259 (2)	
H27A	0.3919	0.8933	0.0871	0.031*	
H27B	0.3653	0.7950	0.0874	0.031*	
C28	0.27154 (9)	0.95937 (7)	0.12110 (8)	0.0266 (2)	
C29	0.17925 (8)	0.97655 (7)	0.11620 (8)	0.0258 (2)	
C30	0.14390 (9)	1.05744 (7)	0.09358 (8)	0.0270 (2)	
H30	0.1778	1.1002	0.0811	0.032*	
C31	0.05758 (9)	1.07557 (7)	0.08930 (8)	0.0261 (2)	
C32	0.00763 (9)	1.01335 (8)	0.10696 (8)	0.0275 (2)	
H32	−0.0511	1.0259	0.1040	0.033*	
C33	0.04495 (9)	0.93180 (8)	0.12925 (9)	0.0292 (3)	
C34	0.13050 (9)	0.91234 (8)	0.13456 (8)	0.0289 (3)	
H34	0.1555	0.8569	0.1502	0.035*	
C35	−0.05544 (9)	1.18186 (8)	0.07035 (9)	0.0312 (3)	
H35A	−0.0516	1.1714	0.1270	0.037*	
H35B	−0.1071	1.1485	0.0297	0.037*	
C36	−0.06892 (9)	1.27525 (8)	0.04970 (8)	0.0289 (3)	
C37	−0.14460 (10)	1.30486 (9)	−0.01773 (9)	0.0365 (3)	
H37	−0.1888	1.2656	−0.0531	0.044*	
C38	−0.15680 (12)	1.39206 (10)	−0.03448 (10)	0.0442 (4)	
H38	−0.2093	1.4118	−0.0810	0.053*	
C39	−0.09368 (13)	1.44910 (10)	0.01558 (12)	0.0481 (4)	
H39	−0.1026	1.5085	0.0044	0.058*	
C40	−0.01739 (14)	1.42040 (10)	0.08203 (13)	0.0566 (5)	
H40	0.0269	1.4600	0.1166	0.068*	
C41	−0.00462 (12)	1.33398 (10)	0.09894 (11)	0.0461 (4)	
H41	0.0488	1.3147	0.1448	0.055*	
C42	0.02282 (11)	0.78935 (8)	0.16259 (10)	0.0371 (3)	
H42A	0.0810	0.7882	0.2135	0.045*	
H42B	0.0341	0.7665	0.1155	0.045*	
C43	−0.04667 (10)	0.73513 (8)	0.17551 (9)	0.0339 (3)	
C44	−0.11661 (11)	0.76979 (10)	0.19029 (9)	0.0387 (3)	
H44	−0.1233	0.8300	0.1907	0.046*	
C45	−0.17766 (12)	0.71686 (12)	0.20469 (10)	0.0495 (4)	
H45	−0.2256	0.7410	0.2152	0.059*	
C46	−0.16855 (14)	0.62960 (12)	0.20376 (11)	0.0551 (5)	
H46	−0.2101	0.5936	0.2138	0.066*	
C48	−0.03825 (13)	0.64694 (9)	0.17448 (11)	0.0465 (4)	
H48	0.0097	0.6224	0.1642	0.056*	
C47	−0.09962 (15)	0.59470 (11)	0.18843 (11)	0.0578 (5)	
H47	−0.0938	0.5345	0.1873	0.069*	

### Atomic displacement parameters (Å^2^)

**Table:**

	*U*^11^	*U*^22^	*U*^33^	*U*^12^	*U*^13^	*U*^23^
O1	0.0326 (5)	0.0186 (3)	0.0293 (4)	−0.0060 (3)	0.0209 (4)	−0.0063 (3)
O2	0.0267 (4)	0.0188 (4)	0.0355 (5)	0.0004 (3)	0.0142 (4)	0.0002 (3)
O3	0.0307 (5)	0.0218 (4)	0.0535 (6)	−0.0023 (4)	0.0177 (5)	0.0007 (4)
O4	0.0326 (5)	0.0187 (4)	0.0493 (6)	0.0035 (3)	0.0231 (4)	0.0048 (4)
O5	0.0388 (6)	0.0226 (4)	0.0617 (7)	0.0014 (4)	0.0303 (5)	0.0103 (4)
N1	0.1162 (15)	0.0314 (6)	0.0732 (10)	−0.0188 (8)	0.0769 (11)	−0.0161 (6)
N2	0.0481 (7)	0.0293 (5)	0.0517 (7)	−0.0109 (5)	0.0333 (6)	−0.0112 (5)
N3	0.0851 (12)	0.0507 (8)	0.0711 (10)	−0.0260 (8)	0.0638 (10)	−0.0172 (7)
N4	0.0282 (5)	0.0182 (4)	0.0264 (5)	−0.0009 (4)	0.0153 (4)	−0.0028 (3)
C1	0.0633 (10)	0.0191 (5)	0.0462 (8)	−0.0076 (6)	0.0387 (8)	−0.0077 (5)
C2	0.0367 (7)	0.0200 (5)	0.0317 (6)	−0.0033 (4)	0.0228 (5)	−0.0039 (4)
C3	0.0359 (7)	0.0235 (5)	0.0322 (6)	−0.0024 (5)	0.0215 (5)	−0.0065 (5)
C4	0.0265 (5)	0.0167 (4)	0.0234 (5)	−0.0004 (4)	0.0133 (4)	0.0010 (4)
C5	0.0277 (6)	0.0163 (4)	0.0244 (5)	−0.0029 (4)	0.0155 (5)	−0.0036 (4)
C6	0.0278 (6)	0.0184 (5)	0.0246 (5)	−0.0010 (4)	0.0155 (5)	−0.0004 (4)
C7	0.0311 (6)	0.0184 (5)	0.0276 (6)	−0.0031 (4)	0.0189 (5)	−0.0021 (4)
C8	0.0351 (7)	0.0226 (5)	0.0262 (6)	−0.0049 (5)	0.0152 (5)	−0.0004 (4)
C9	0.0284 (6)	0.0270 (6)	0.0394 (7)	−0.0004 (5)	0.0172 (6)	−0.0067 (5)
C10	0.0480 (8)	0.0256 (6)	0.0402 (7)	−0.0083 (5)	0.0311 (7)	−0.0070 (5)
C11	0.0306 (6)	0.0180 (5)	0.0264 (5)	−0.0019 (4)	0.0162 (5)	0.0008 (4)
C12	0.0273 (6)	0.0172 (5)	0.0244 (5)	−0.0009 (4)	0.0123 (5)	0.0013 (4)
C13	0.0289 (6)	0.0169 (5)	0.0263 (5)	−0.0008 (4)	0.0139 (5)	0.0008 (4)
C14	0.0264 (6)	0.0182 (5)	0.0252 (5)	−0.0009 (4)	0.0120 (5)	0.0015 (4)
C15	0.0277 (6)	0.0172 (5)	0.0271 (6)	−0.0020 (4)	0.0154 (5)	−0.0007 (4)
C16	0.0245 (5)	0.0184 (5)	0.0241 (5)	−0.0011 (4)	0.0122 (4)	−0.0004 (4)
C17	0.0308 (6)	0.0268 (5)	0.0253 (6)	−0.0018 (5)	0.0161 (5)	0.0006 (4)
C18	0.0349 (7)	0.0341 (6)	0.0284 (6)	0.0024 (5)	0.0186 (5)	−0.0029 (5)
C19	0.0545 (10)	0.0544 (9)	0.0395 (8)	0.0036 (8)	0.0328 (8)	−0.0041 (7)
C20	0.0636 (11)	0.0609 (11)	0.0450 (9)	0.0115 (9)	0.0343 (9)	−0.0126 (8)
C21	0.0510 (9)	0.0424 (8)	0.0415 (8)	0.0117 (7)	0.0184 (7)	−0.0148 (7)
C22	0.0363 (7)	0.0274 (6)	0.0353 (7)	0.0034 (5)	0.0130 (6)	−0.0089 (5)
C23	0.0282 (6)	0.0264 (5)	0.0261 (6)	0.0028 (5)	0.0126 (5)	−0.0055 (4)
C24	0.0419 (8)	0.0343 (7)	0.0269 (6)	−0.0010 (6)	0.0146 (6)	0.0046 (5)
C25	0.0316 (7)	0.0403 (7)	0.0361 (7)	−0.0061 (6)	0.0194 (6)	−0.0007 (6)
C26	0.0266 (6)	0.0174 (5)	0.0319 (6)	−0.0024 (4)	0.0160 (5)	−0.0005 (4)
C27	0.0304 (6)	0.0211 (5)	0.0312 (6)	0.0016 (4)	0.0180 (5)	0.0015 (4)
C28	0.0289 (6)	0.0189 (5)	0.0314 (6)	0.0007 (4)	0.0121 (5)	0.0021 (4)
C29	0.0268 (6)	0.0209 (5)	0.0303 (6)	−0.0012 (4)	0.0128 (5)	−0.0005 (4)
C30	0.0282 (6)	0.0196 (5)	0.0346 (6)	−0.0022 (4)	0.0147 (5)	−0.0004 (4)
C31	0.0309 (6)	0.0174 (5)	0.0319 (6)	0.0004 (4)	0.0150 (5)	0.0001 (4)
C32	0.0284 (6)	0.0225 (5)	0.0350 (6)	−0.0001 (4)	0.0169 (5)	0.0003 (5)
C33	0.0339 (7)	0.0212 (5)	0.0372 (7)	−0.0014 (5)	0.0194 (6)	0.0024 (5)
C34	0.0311 (6)	0.0199 (5)	0.0372 (7)	0.0006 (4)	0.0159 (5)	0.0037 (5)
C35	0.0332 (7)	0.0242 (5)	0.0424 (7)	0.0034 (5)	0.0220 (6)	0.0025 (5)
C36	0.0329 (6)	0.0235 (5)	0.0344 (6)	0.0043 (5)	0.0183 (5)	0.0020 (5)
C37	0.0329 (7)	0.0334 (7)	0.0404 (8)	0.0036 (5)	0.0127 (6)	−0.0024 (6)
C38	0.0465 (9)	0.0405 (8)	0.0414 (8)	0.0166 (7)	0.0144 (7)	0.0096 (6)
C39	0.0593 (10)	0.0267 (7)	0.0609 (10)	0.0097 (7)	0.0279 (9)	0.0078 (7)
C40	0.0586 (11)	0.0271 (7)	0.0676 (12)	−0.0023 (7)	0.0107 (9)	−0.0053 (7)
C41	0.0470 (9)	0.0297 (7)	0.0457 (9)	0.0046 (6)	0.0043 (7)	0.0008 (6)
C42	0.0417 (8)	0.0227 (6)	0.0523 (9)	0.0014 (5)	0.0252 (7)	0.0075 (6)
C43	0.0406 (8)	0.0279 (6)	0.0305 (6)	−0.0057 (5)	0.0123 (6)	0.0057 (5)
C44	0.0418 (8)	0.0366 (7)	0.0378 (7)	−0.0072 (6)	0.0169 (6)	0.0064 (6)
C45	0.0461 (9)	0.0617 (10)	0.0408 (8)	−0.0168 (8)	0.0184 (7)	0.0074 (7)
C46	0.0626 (12)	0.0576 (10)	0.0387 (8)	−0.0318 (9)	0.0154 (8)	0.0090 (7)
C48	0.0629 (11)	0.0283 (7)	0.0469 (9)	−0.0062 (7)	0.0220 (8)	0.0048 (6)
C47	0.0840 (14)	0.0348 (8)	0.0465 (10)	−0.0225 (9)	0.0198 (10)	0.0067 (7)

### Geometric parameters (Å, º)

**Table:**

O1—C6	1.3294 (13)	C21—H21	0.9500
O1—C5	1.4772 (13)	C22—C23	1.3873 (17)
O2—C28	1.3550 (14)	C22—H22	0.9500
O2—C27	1.4387 (15)	C24—H24A	0.9800
O3—C28	1.2036 (15)	C24—H24B	0.9800
O4—C31	1.3686 (14)	C24—H24C	0.9800
O4—C35	1.4344 (16)	C25—H25A	0.9800
O5—C33	1.3694 (15)	C25—H25B	0.9800
O5—C42	1.4215 (15)	C25—H25C	0.9800
N1—C1	1.1433 (18)	C26—C27	1.5128 (18)
N2—C3	1.1510 (16)	C26—H26A	0.9900
N3—C10	1.1446 (18)	C26—H26B	0.9900
N4—C16	1.3570 (14)	C27—H27A	0.9900
N4—C23	1.4119 (15)	C27—H27B	0.9900
N4—C26	1.4615 (15)	C28—C29	1.4845 (18)
C1—C2	1.4170 (17)	C29—C30	1.3793 (17)
C2—C6	1.3830 (16)	C29—C34	1.3994 (17)
C2—C3	1.4187 (17)	C30—C31	1.3968 (18)
C4—C11	1.3945 (15)	C30—H30	0.9500
C4—C7	1.3947 (15)	C31—C32	1.3843 (17)
C4—C5	1.5173 (15)	C32—C33	1.3979 (17)
C5—C9	1.5170 (17)	C32—H32	0.9500
C5—C8	1.5182 (17)	C33—C34	1.3823 (18)
C6—C7	1.4190 (15)	C34—H34	0.9500
C7—C10	1.4162 (17)	C35—C36	1.5025 (17)
C8—H8A	0.9800	C35—H35A	0.9900
C8—H8B	0.9800	C35—H35B	0.9900
C8—H8C	0.9800	C36—C37	1.378 (2)
C9—H9A	0.9800	C36—C41	1.386 (2)
C9—H9B	0.9800	C37—C38	1.395 (2)
C9—H9C	0.9800	C37—H37	0.9500
C11—C12	1.3854 (16)	C38—C39	1.364 (3)
C11—H11	0.9500	C38—H38	0.9500
C12—C13	1.3959 (15)	C39—C40	1.370 (3)
C12—H12	0.9500	C39—H39	0.9500
C13—C14	1.3801 (16)	C40—C41	1.384 (2)
C13—H13	0.9500	C40—H40	0.9500
C14—C15	1.3969 (15)	C41—H41	0.9500
C14—H14	0.9500	C42—C43	1.5027 (19)
C15—C16	1.3826 (15)	C42—H42A	0.9900
C15—H15	0.9500	C42—H42B	0.9900
C16—C17	1.5328 (16)	C43—C44	1.377 (2)
C17—C18	1.5124 (18)	C43—C48	1.391 (2)
C17—C25	1.5367 (19)	C44—C45	1.394 (2)
C17—C24	1.5387 (18)	C44—H44	0.9500
C18—C19	1.3845 (19)	C45—C46	1.377 (3)
C18—C23	1.3846 (19)	C45—H45	0.9500
C19—C20	1.392 (2)	C46—C47	1.369 (3)
C19—H19	0.9500	C46—H46	0.9500
C20—C21	1.376 (3)	C48—C47	1.388 (2)
C20—H20	0.9500	C48—H48	0.9500
C21—C22	1.395 (2)	C47—H47	0.9500
			
C6—O1—C5	110.42 (8)	C17—C25—H25A	109.5
C28—O2—C27	116.33 (9)	C17—C25—H25B	109.5
C31—O4—C35	117.11 (10)	H25A—C25—H25B	109.5
C33—O5—C42	116.65 (11)	C17—C25—H25C	109.5
C16—N4—C23	111.27 (10)	H25A—C25—H25C	109.5
C16—N4—C26	125.20 (9)	H25B—C25—H25C	109.5
C23—N4—C26	123.17 (9)	N4—C26—C27	112.24 (10)
N1—C1—C2	176.93 (18)	N4—C26—H26A	109.2
C6—C2—C1	121.67 (11)	C27—C26—H26A	109.2
C6—C2—C3	121.70 (11)	N4—C26—H26B	109.2
C1—C2—C3	116.52 (11)	C27—C26—H26B	109.2
N2—C3—C2	176.49 (13)	H26A—C26—H26B	107.9
C11—C4—C7	125.42 (10)	O2—C27—C26	111.36 (10)
C11—C4—C5	127.58 (10)	O2—C27—H27A	109.4
C7—C4—C5	106.97 (9)	C26—C27—H27A	109.4
O1—C5—C9	106.09 (9)	O2—C27—H27B	109.4
O1—C5—C4	102.95 (8)	C26—C27—H27B	109.4
C9—C5—C4	113.33 (10)	H27A—C27—H27B	108.0
O1—C5—C8	105.78 (9)	O3—C28—O2	123.14 (12)
C9—C5—C8	113.60 (10)	O3—C28—C29	125.68 (11)
C4—C5—C8	113.84 (10)	O2—C28—C29	111.18 (10)
O1—C6—C2	118.73 (10)	C30—C29—C34	121.53 (12)
O1—C6—C7	110.45 (9)	C30—C29—C28	117.90 (11)
C2—C6—C7	130.82 (11)	C34—C29—C28	120.57 (11)
C4—C7—C10	124.33 (11)	C29—C30—C31	119.08 (11)
C4—C7—C6	109.18 (10)	C29—C30—H30	120.5
C10—C7—C6	126.50 (11)	C31—C30—H30	120.5
C5—C8—H8A	109.5	O4—C31—C32	124.20 (11)
C5—C8—H8B	109.5	O4—C31—C30	115.07 (10)
H8A—C8—H8B	109.5	C32—C31—C30	120.73 (11)
C5—C8—H8C	109.5	C31—C32—C33	118.94 (12)
H8A—C8—H8C	109.5	C31—C32—H32	120.5
H8B—C8—H8C	109.5	C33—C32—H32	120.5
C5—C9—H9A	109.5	O5—C33—C34	123.86 (11)
C5—C9—H9B	109.5	O5—C33—C32	114.65 (11)
H9A—C9—H9B	109.5	C34—C33—C32	121.49 (11)
C5—C9—H9C	109.5	C33—C34—C29	118.22 (11)
H9A—C9—H9C	109.5	C33—C34—H34	120.9
H9B—C9—H9C	109.5	C29—C34—H34	120.9
N3—C10—C7	177.74 (15)	O4—C35—C36	107.01 (10)
C12—C11—C4	125.74 (11)	O4—C35—H35A	110.3
C12—C11—H11	117.1	C36—C35—H35A	110.3
C4—C11—H11	117.1	O4—C35—H35B	110.3
C11—C12—C13	124.47 (11)	C36—C35—H35B	110.3
C11—C12—H12	117.8	H35A—C35—H35B	108.6
C13—C12—H12	117.8	C37—C36—C41	118.36 (13)
C14—C13—C12	121.48 (11)	C37—C36—C35	121.51 (13)
C14—C13—H13	119.3	C41—C36—C35	120.13 (13)
C12—C13—H13	119.3	C36—C37—C38	120.50 (14)
C13—C14—C15	123.46 (11)	C36—C37—H37	119.8
C13—C14—H14	118.3	C38—C37—H37	119.8
C15—C14—H14	118.3	C39—C38—C37	120.35 (15)
C16—C15—C14	125.44 (11)	C39—C38—H38	119.8
C16—C15—H15	117.3	C37—C38—H38	119.8
C14—C15—H15	117.3	C38—C39—C40	119.74 (14)
N4—C16—C15	121.99 (10)	C38—C39—H39	120.1
N4—C16—C17	108.97 (9)	C40—C39—H39	120.1
C15—C16—C17	129.03 (10)	C39—C40—C41	120.29 (16)
C18—C17—C16	101.06 (9)	C39—C40—H40	119.9
C18—C17—C25	110.12 (11)	C41—C40—H40	119.9
C16—C17—C25	112.71 (10)	C40—C41—C36	120.74 (15)
C18—C17—C24	110.74 (11)	C40—C41—H41	119.6
C16—C17—C24	110.46 (11)	C36—C41—H41	119.6
C25—C17—C24	111.32 (11)	O5—C42—C43	109.42 (12)
C19—C18—C23	119.80 (13)	O5—C42—H42A	109.8
C19—C18—C17	130.34 (13)	C43—C42—H42A	109.8
C23—C18—C17	109.85 (10)	O5—C42—H42B	109.8
C18—C19—C20	118.48 (15)	C43—C42—H42B	109.8
C18—C19—H19	120.8	H42A—C42—H42B	108.2
C20—C19—H19	120.8	C44—C43—C48	119.21 (14)
C21—C20—C19	120.91 (14)	C44—C43—C42	122.29 (13)
C21—C20—H20	119.5	C48—C43—C42	118.48 (14)
C19—C20—H20	119.5	C43—C44—C45	120.21 (15)
C20—C21—C22	121.59 (14)	C43—C44—H44	119.9
C20—C21—H21	119.2	C45—C44—H44	119.9
C22—C21—H21	119.2	C46—C45—C44	120.10 (18)
C23—C22—C21	116.54 (14)	C46—C45—H45	119.9
C23—C22—H22	121.7	C44—C45—H45	119.9
C21—C22—H22	121.7	C47—C46—C45	120.00 (16)
C18—C23—C22	122.67 (12)	C47—C46—H46	120.0
C18—C23—N4	108.78 (10)	C45—C46—H46	120.0
C22—C23—N4	128.55 (12)	C47—C48—C43	120.21 (18)
C17—C24—H24A	109.5	C47—C48—H48	119.9
C17—C24—H24B	109.5	C43—C48—H48	119.9
H24A—C24—H24B	109.5	C46—C47—C48	120.27 (17)
C17—C24—H24C	109.5	C46—C47—H47	119.9
H24A—C24—H24C	109.5	C48—C47—H47	119.9
H24B—C24—H24C	109.5		
			
C6—O1—C5—C9	120.87 (11)	C17—C18—C23—N4	−0.33 (15)
C6—O1—C5—C4	1.58 (12)	C21—C22—C23—C18	0.3 (2)
C6—O1—C5—C8	−118.16 (10)	C21—C22—C23—N4	−179.88 (13)
C11—C4—C5—O1	177.18 (11)	C16—N4—C23—C18	1.80 (14)
C7—C4—C5—O1	−1.19 (12)	C26—N4—C23—C18	175.25 (11)
C11—C4—C5—C9	63.04 (16)	C16—N4—C23—C22	−178.07 (13)
C7—C4—C5—C9	−115.32 (11)	C26—N4—C23—C22	−4.6 (2)
C11—C4—C5—C8	−68.82 (15)	C16—N4—C26—C27	72.97 (14)
C7—C4—C5—C8	112.81 (11)	C23—N4—C26—C27	−99.56 (13)
C5—O1—C6—C2	178.65 (11)	C28—O2—C27—C26	80.57 (12)
C5—O1—C6—C7	−1.38 (13)	N4—C26—C27—O2	45.16 (12)
C1—C2—C6—O1	173.19 (13)	C27—O2—C28—O3	0.31 (18)
C3—C2—C6—O1	−2.79 (19)	C27—O2—C28—C29	−178.92 (10)
C1—C2—C6—C7	−6.8 (2)	O3—C28—C29—C30	24.9 (2)
C3—C2—C6—C7	177.25 (13)	O2—C28—C29—C30	−155.89 (11)
C11—C4—C7—C10	1.9 (2)	O3—C28—C29—C34	−154.74 (14)
C5—C4—C7—C10	−179.64 (13)	O2—C28—C29—C34	24.47 (17)
C11—C4—C7—C6	−177.95 (12)	C34—C29—C30—C31	0.28 (19)
C5—C4—C7—C6	0.46 (14)	C28—C29—C30—C31	−179.36 (11)
O1—C6—C7—C4	0.56 (14)	C35—O4—C31—C32	7.28 (18)
C2—C6—C7—C4	−179.47 (13)	C35—O4—C31—C30	−173.09 (11)
O1—C6—C7—C10	−179.33 (13)	C29—C30—C31—O4	179.96 (11)
C2—C6—C7—C10	0.6 (2)	C29—C30—C31—C32	−0.39 (19)
C7—C4—C11—C12	−179.67 (12)	O4—C31—C32—C33	179.65 (12)
C5—C4—C11—C12	2.2 (2)	C30—C31—C32—C33	0.03 (19)
C4—C11—C12—C13	−174.28 (12)	C42—O5—C33—C34	−3.8 (2)
C11—C12—C13—C14	177.11 (12)	C42—O5—C33—C32	176.13 (12)
C12—C13—C14—C15	−171.91 (12)	C31—C32—C33—O5	−179.50 (12)
C13—C14—C15—C16	173.53 (12)	C31—C32—C33—C34	0.5 (2)
C23—N4—C16—C15	177.74 (11)	O5—C33—C34—C29	179.39 (12)
C26—N4—C16—C15	4.45 (18)	C32—C33—C34—C29	−0.6 (2)
C23—N4—C16—C17	−2.48 (14)	C30—C29—C34—C33	0.2 (2)
C26—N4—C16—C17	−175.77 (11)	C28—C29—C34—C33	179.82 (12)
C14—C15—C16—N4	−175.51 (12)	C31—O4—C35—C36	175.24 (11)
C14—C15—C16—C17	4.8 (2)	O4—C35—C36—C37	119.95 (14)
N4—C16—C17—C18	2.10 (13)	O4—C35—C36—C41	−60.16 (17)
C15—C16—C17—C18	−178.15 (12)	C41—C36—C37—C38	−1.5 (2)
N4—C16—C17—C25	119.60 (12)	C35—C36—C37—C38	178.39 (14)
C15—C16—C17—C25	−60.65 (17)	C36—C37—C38—C39	0.3 (2)
N4—C16—C17—C24	−115.17 (11)	C37—C38—C39—C40	0.8 (3)
C15—C16—C17—C24	64.58 (16)	C38—C39—C40—C41	−0.6 (3)
C16—C17—C18—C19	177.87 (16)	C39—C40—C41—C36	−0.7 (3)
C25—C17—C18—C19	58.5 (2)	C37—C36—C41—C40	1.7 (3)
C24—C17—C18—C19	−65.1 (2)	C35—C36—C41—C40	−178.20 (17)
C16—C17—C18—C23	−1.03 (14)	C33—O5—C42—C43	−178.33 (12)
C25—C17—C18—C23	−120.40 (12)	O5—C42—C43—C44	−16.2 (2)
C24—C17—C18—C23	116.04 (12)	O5—C42—C43—C48	165.43 (14)
C23—C18—C19—C20	−0.9 (2)	C48—C43—C44—C45	0.5 (2)
C17—C18—C19—C20	−179.73 (16)	C42—C43—C44—C45	−177.84 (14)
C18—C19—C20—C21	0.6 (3)	C43—C44—C45—C46	−0.3 (2)
C19—C20—C21—C22	0.2 (3)	C44—C45—C46—C47	−0.2 (3)
C20—C21—C22—C23	−0.6 (2)	C44—C43—C48—C47	−0.2 (2)
C19—C18—C23—C22	0.5 (2)	C42—C43—C48—C47	178.25 (15)
C17—C18—C23—C22	179.55 (12)	C45—C46—C47—C48	0.5 (3)
C19—C18—C23—N4	−179.36 (13)	C43—C48—C47—C46	−0.4 (3)

### Hydrogen-bond geometry (Å, º)

Cg1 is the centroid of the C29–C34 ring.

**Table:**

*D*—H···*A*	*D*—H	H···*A*	*D*···*A*	*D*—H···*A*
C15—H15···N3^i^	0.95	2.48	3.404 (2)	165
C30—H30···O1^ii^	0.95	2.50	3.4136 (17)	162
C32—H32···N2^iii^	0.95	2.54	3.475 (2)	167
C9—H9*A*···*Cg*1^iii^	0.98	2.89	3.8454 (16)	166

Symmetry codes: (i) −*x*+1, −*y*+1, −*z*; (ii) *x*, *y*+1, *z*; (iii) −*x*, −*y*+1, −*z*.
